# BODE index versus GOLD classification for explaining anxious and depressive symptoms in patients with COPD – a cross-sectional study

**DOI:** 10.1186/1465-9921-10-1

**Published:** 2009-01-09

**Authors:** Georg-Christian Funk, Kathrin Kirchheiner, Otto Chris Burghuber, Sylvia Hartl

**Affiliations:** 1 Department of Respiratory and Critical Care Medicine and Ludwig Boltzmann Institute for Chronic Obstructive Pulmonary Disease, Otto Wagner Hospital, Vienna, Austria

## Abstract

**Background:**

Anxiety and depression are common and treatable risk factors for re-hospitalisation and death in patients with COPD. The degree of lung function impairment does not sufficiently explain anxiety and depression. The BODE index allows a functional classification of COPD beyond FEV_1_. The aim of this cross-sectional study was (1) to test whether the BODE index is superior to the GOLD classification for explaining anxious and depressive symptoms; and (2) to assess which components of the BODE index are associated with these psychological aspects of COPD.

**Methods:**

COPD was classified according to the GOLD stages based on FEV_1%predicted _in 122 stable patients with COPD. An additional four stage classification was constructed based on the quartiles of the BODE index. The hospital anxiety and depression scale was used to assess anxious and depressive symptoms.

**Results:**

The overall prevalence of anxious and depressive symptoms was 49% and 52%, respectively. The prevalence of anxious symptoms increased with increasing BODE stages but not with increasing GOLD stages. The prevalence of depressive symptoms increased with both increasing GOLD and BODE stages. The BODE index was superior to FEV_1%predicted _for explaining anxious and depressive symptoms. Anxious symptoms were explained by dyspnoea. Depressive symptoms were explained by both dyspnoea and reduced exercise capacity.

**Conclusion:**

The BODE index is superior to the GOLD classification for explaining anxious and depressive symptoms in COPD patients. These psychological consequences of the disease may play a role in future classification systems of COPD.

## Background

Chronic obstructive pulmonary disease (COPD) is a progressive disorder leading to substantial mortality and morbidity. Treatment goals in COPD are prevention or deceleration of progression and increasing patients' quality of life [1]. Apart from physical impairment, patients with COPD carry substantial mental burden related to their disease and its symptoms. Patients frequently suffer from anxiety [2-7] and depression [2-10]. Both anxiety and depression are risk factors for rehospitalisation in COPD [6,7]. Co-morbid depression is associated with longer hospitalisation stay and poorer survival [9]. Analogously to congestive heart failure [11-14], coronary artery disease [15] and diabetes [16] psychological disorders are becoming increasingly recognized as important outcome-modifying co-morbidities in COPD. Irrespective of somatic diseases, anxiety and depression themselves are risk factors of increased mortality [17-19]. While the mechanisms of these associations are largely unknown, they are susceptible to therapeutic intervention; treating major depression in older patients decreases their mortality [20,21].

Whether the severity of the lung function impairment is related to anxiety and depression in patients with COPD has been subject of research. In most studies FEV_1 _was a bad predictor of anxiety and depression [2,7,9,10,22].

On the other hand, the presence of respiratory symptoms causes substantial anxiety and depression [23]. Dyspnoea has been shown to correlate with anxiety and depression in patients with COPD [22]. The BODE index (body mass index, airflow obstruction, dyspnoea, and exercise capacity) is a multistage functional scoring system for COPD comprising an assessment of symptoms, a surrogate of the nutritional state, and exercise capacity together with the spirometric measure of airflow (FEV_1_) [24]. This multidimensional grading system was shown to be superior over the FEV_1_-based GOLD classification [25] for predicting hospitalization and the risk of death among patients with COPD [24,26]. Given the incorporation of the subjective variable 'dyspnoea' and the individual exercise capacity, the BODE index should be closer related to the individual subjective consequences of COPD than lung function alone.

The aim of this study was twofold. First, to test whether the BODE index is superior to the GOLD classification for explaining of anxious and depressive symptoms. Second, to assess which components of the BODE index are associated with these psychological aspects of COPD.

## Patients and methods

### Patient recruitment

This was a prospective cross-sectional study performed at the Department of Respiratory and Critical Care Medicine of a primary hospital in Vienna between January 2006 and May 2007. Adult (≥ 18 yr) in- and out-patients of the institution were screened for the study. The study was approved by the Institutional ethics committee and written informed consent was obtained from all patients.

### Inclusion and exclusion criteria

Inclusion criteria were (1) COPD diagnosed according to the GOLD consensus [25], (2) Stable conditions i.e. absence of exacerbation (patients could be recruited during exacerbations but were investigated after a stable period of at least 3 months), (3) ability to perform a six minute walking test.

Exclusion criteria were (1) absence of informed consent, (2) insufficient knowledge of German for completing the questionnaires, (3) unstable coronary artery disease, (4) history of congestive heart failure, (5) significant pulmonary disease other than COPD (e.g. asthma or lung cancer), (6) significant neurological disease.

All together 228 patients were screened, of which 151 were eligible according to the inclusion and exclusion criteria. Of those 122 patients agreed to participate in the study (response rate 81%).

### Classification of COPD

Spirometry was performed according to the ATS/ERS recommendations [27] using a standard PFT unit (SensorMedics Vmax 22, Viasys Healthcare). Blood gases were determined in arterialised ear lobe samples using the AVL Compact 3 Blood Gas Analyzer (Roche Diagnostics, Graz, Austria). COPD was classified according to the guidelines of the Global Initiative for Obstructive Lung Disease (GOLD).

Additionally the BODE index was calculated for classification of COPD. The score comprises body mass index (BMI), post-bronchodilator FEV_1%predicted_, grade of dyspnoea (measured by the modified Medical Research Council dyspnoea scale, MMRC) and the six-minute-walking-distance [24]. For calculation of the BODE index, we used the empirical model as previously described [24]: for each threshold value of FEV_1%predicted_, distance walked in six minutes, and score on the MMRC dyspnoea scale [28], the patients received points ranging from 0 (lowest value) to 3 (maximal value). For body mass index the values were 0 or 1. The points for each variable were added, so that the BODE index ranged from 0 to 10 points in each patient. The post bronchodilator FEV_1%predicted _was used and classified according to the three stages identified by the American Thoracic Society [29]. The best of two 6-min walk tests performed at least 30-min apart [30] was taken as a surrogate of exercise capacity and was used for scoring. Variables and point values used for the computation of the BODE index are shown in table 1. Finally after obtaining the BODE index for all patients, quartiles of the BODE index were used to construct four severity stages [24,26]:

**Table 1:** Variables and Point Values Used for the Computation of the Body-Mass Index, Degree of Airflow Obstruction and Dyspnoea, and Exercise Capacity (BODE) Index according to [24].*

Variable	Points on the BODE Index			
	0	1	2	3

FEV_1%predicted _†	≥ 65	50–64	36–49	≤ 35

Distance walked in 6 min (m)	≥ 350	250–349	150–249	≤ 149

MMRC dyspnoea scale‡	0–1	2	3	4

Body mass index§	>21	≤ 21		

* The cut-off values for the assignment of points are shown for each variable. The total possible values range from 0 to 10. FEV_1%predicted _denotes forced expiratory volume in one second as a percentage of the predicted value.

† The FEV_1%predicted _categories are based on stages identified by the American Thoracic Society.

‡ Scores on the modified Medical Research Council (MMRC) dyspnoea scale can range from 0 to 4; 0 – "Not troubled with breathlessness except with strenuous exercise"; 1 – "Troubled by shortness of breath when hurrying on the level or walking up a slight hill"; 2 – "Walks slower than people of the same age on the level because of breathlessness or has to stop for breath when walking at own pace on the level"; 3 – "Stops for breath after walking about 100 yards or after a few minutes on the level"; 4 – "Too breathless to leave the house or breathless when dressing or undressing"

§ The values for body-mass index were 0 or 1 because of the inflection point in the inverse relation between survival and body-mass index at a value of 21.

BODE stage I = BODE index 0 – 2;

BODE stage II = BODE index 3 and 4;

BODE stage III = BODE index 5 – 7;

BODE stage IV = BODE index 8 – 10.

### Questionnaires

The self-reported hospital anxiety and depression (HAD) scale was used to screen for psychiatric co-morbidity. The HAD scale is a validated tool for detecting psychiatric co-morbidity in patients with somatic disease. It has previously been applied to COPD patients [2,5-7,9,22]. The HAD scale consists of seven questions related to anxiety and seven questions related to depression. Each item is rated on a 4-point scale, yielding maximum subscale scores of 21 for anxiety (anxiety score) and depression (depression score), respectively. Scores on either subscale of ≥ 8 describe the presence of symptoms suggestive of depression or anxiety, respectively [6,7,9,31]. The HAD scale is a screening tool for anxiety and depression but does not allow a diagnosis of anxiety and depression to be made.

### Statistics

Data on interval scales were described by means± standard deviations, data on ordinal scales by medians (1^st ^to 3^rd ^quartiles). Normality was assessed using normal plots and data were transformed as needed. Differences between means were tested with Student's t-test and reported with 95% confidence intervals (95%CI). Differences of the anxiety score and the depression score between the different stages of disease severity were tested for by one-way ANOVA. Categorical variables were described by frequencies and percentages. Differences of proportions between COPD or BODE stages were compared by the χ^2 ^test for trend. Correlation between ordinal and interval data was determined by Kendall's rank correlation. Linear regression was used to determine which components of the BODE were independently associated with the psychological scores. FEV_1%predicted _and BMI were logarithm transformed prior to entry into linear regression. Collinearity was controlled by means of the variance inflation factor. Statistics were performed by SPSS 15.0 (Chicago, IL). Significance was accepted at p < 0.05.

## Results

### Patient characteristics

One hundred twenty two patients were included in the study. The baseline characteristics of these patients are shown in table 2. The number of patients in stages I to IV of COPD severity as defined by GOLD and the median BODE index of the patients in each stage are shown in table 3. The majority of patients had severe-to-very severe COPD (stages III to IV). The median BODE index increased from stage I to stage IV.

**Table 2:** Baseline characteristics of the patients sample (n = 122)*

Characteristics	Data
Male/female gender, N/N	68/54

Age, yr	65 ± 10

Body mass index, kg/m^2^	25.8 ± 6.8

FEV_1_, liters	1.2 ± 0.6

FEV_1%predicted_	44.5 ± 19.3

FVC, liters	2.6 ± 0.9

FEV_1_/FVC	44.8 ± 11.9

paO_2_, mmHg	64.0 ± 9.7

paCO_2_, mmHg	40.6 ± 5.7

Modified MRC dyspnoea scale	1.9 ± 1.3

Six-minute walking distance, meter	303 ± 140

BODE index	4.3 ± 2.8

*Values are presented as mean ± standard deviation unless otherwise indicated.

**Table 3:** Classification of patients according to GOLD with the BODE index in each stage (n = 122); the BODE index is given as median and 1^st ^to 3^rd ^quartiles.

Severity of COPD according to GOLD	Patients, N (%)	BODE index
Stage I (FEV_1 _≥ 80% predicted)	6 (5)	0 (0 to 0)

Stage II (50% ≤ FEV_1 _< 80% predicted)	39 (32)	1 (1 to 4)

Stage III (30% ≤ FEV_1 _< 50% predicted)	31 (25)	4 (3 to 6)

Stage IV (FEV1 ≤ 30% predicted)	46 (38)	7 (5 to 8)

### Symptoms of anxiety and depression

The mean anxiety score and the mean depression score were 8.0 ± 4.3 and 7.8 ± 4.5, respectively. 60 patients (49%) and 63 patients (52%) were found to have symptoms suggestive of anxiety and depression, respectively. Anxious symptoms were more common in women (59% in women versus 41% in men, *p *= 0.036). Presence of depressive symptoms was independent of gender (51% of the men; 52% of the women). FEV_1%predicted _was lower in patients with anxious symptoms (40.5 ± 17.3) compared to patients without (48.3 ± 20.5), mean difference -7.8, 95%CI -14.5 to -1.1; *p *= 0.025. FEV_1%predicted _was lower in patients with depressive symptoms (37.0 ± 15.2) compared to patients without (52.5 ± 20.0), mean difference -15.5, 95%CI -21.8 to -9.2; p < 0.001. 78% of the patients with anxious symptoms also had depressive symptoms and 75% of the patients with depressive symptoms also had anxious symptoms.

### Anxiety and depression in COPD classified by GOLD or BODE

**Figure 1:**
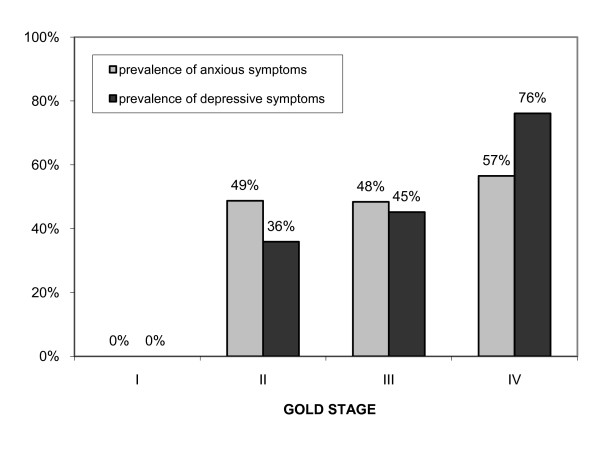
**Prevalence of anxious and depressive symptoms in patients with COPD classified according to GOLD stages**.

**Figure 2:**
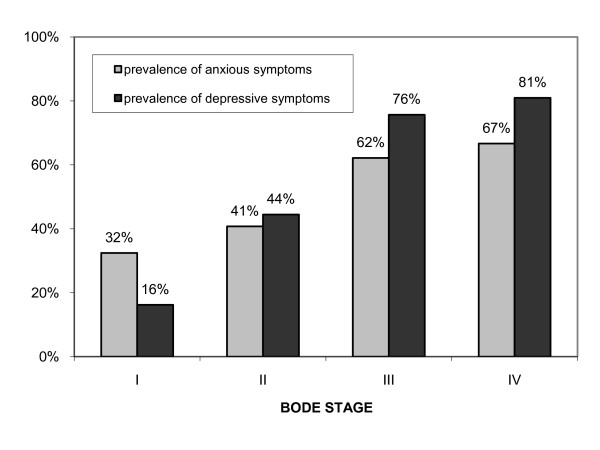
**Prevalence of anxious and depressive symptoms in patients with COPD classified according to quartiles of the BODE index**.

The anxiety score and the depression score correlated closer with the BODE index (Kτ = 0.20, *p *= 0.001; Kτ = 0.41, *p *< 0.001; respectively) than with FEV_1%predicted _(Kτ = -0.13, *p *= 0.037; Kτ = -0.28, *p *< 0.001; respectively). The prevalence of anxiety increased with increasing BODE stage (χ^2 ^= 9.38, *p *= 0.002) but not with increasing GOLD stages (χ^2 ^= 3.29, *p *= 0.070). The prevalence of depression increased with both increasing GOLD and BODE stages (χ^2 ^= 20.47, *p *< 0.001; χ^2 ^= 32.84, *p *< 0.001). The prevalences of anxious and depressive symptoms within the GOLD and BODE stages are shown in Figures 1 and 2.

The mean anxiety score in the GOLD stages I, II, III and IV was 3.7 ± 2.6, 7.9 ± 4.2, 8.0 ± 4.1 and 8.6 ± 4.2, respectively; p = 0.069. The mean depression score in the GOLD stages I, II, III and IV was 1.5 ± 1.4, 6.7 ± 4.6, 7.8 ± 4.7 and 9.3 ± 4.5, respectively; p < 0.0001. The mean anxiety score in the BODE stages I, II, III and IV was 6.3 ± 3.5, 7.7 ± 4.6, 9.5 ± 4.1 and 8.5 ± 4.6, respectively; p = 0.009. The mean depression score in the BODE stages I, II, III and IV was 4.6 ± 3.1, 7.2 ± 4.3, 9.7 ± 3.4 and 10.9 ± 4.2, respectively; p < 0.0001.

### Association of the components of the BODE index with anxiety and depression

Linear regression was used to determine which components of the BODE index were independently associated with the anxiety and depression score. The six minute walking distance and the MMRC dyspnoea scale were independently associated with the depression score, whereas the MMRC dyspnoea scale had a borderline significant association with the anxiety score. (Table 4). After removing the non-significant BMI and FEV_1%predicted _from the regression equation and adjusting for the six-minute walking distance the MMRC dyspnoea scale was significantly associated with the anxiety score (MMRC dyspnoea scale: β = 0.75, *p *= 0.043; six-minute walking distance: β = -0.002, *p *= 0.497). FEV_1%predicted _and BMI were associated with neither anxiety nor depression.

**Table 4:** Linear regression of the components of the BODE index on the anxiety score and the depression score.

	FEV_1%predicted _*		6 minute walking distance		body mass index*		MMRC dyspnoea score	
	β	*p*	β	*p*	β	*p*	β	*p*

anxiety score	-0.030	0.787	-0.083	0.472	0.126	0.167	0.222	0.067

depression score	-0.090	0.346	-0.338	0.001	-0.016	0.837	0.227	0.031

* FEV_1%predicted _and Body mass index were logarithm transformed prior to regression

## Discussion

This study demonstrates that anxious and depressive symptoms are common in patients with advanced COPD. The BODE index is superior to the GOLD classification for explaining these symptoms. Anxious symptoms were explained by dyspnoea. Depressive symptoms were explained by both dyspnoea and reduced exercise capacity.

COPD is increasingly considered as a disease not only of the lungs. It has been suggested as a part of the 'chronic systemic inflammatory syndrome' together with the metabolic syndrome, coronary artery disease and others [32]. The complexity of COPD and its frequent co-morbidities requires assessment and staging of the disease beyond the degree of airflow limitation. Using the hospital anxiety and depression score previous studies have yielded prevalences of anxious and depressive symptoms of up to 41% and 44%, respectively in patients with COPD [6,9]. Our findings confirm that both anxious and depressive symptoms are common in COPD and increase with disease severity. The higher prevalence of anxious symptoms in women is a known finding. Female COPD patients were reported to suffer from psychiatric disorders and psychological distress more often than male patients [33].

We found that the degree of lung function impairment cannot sufficiently explain anxious and depressive symptoms in COPD. This is in concordance with previous research. FEV_1%predicted _was similar in patients with anxiety or depression compared to patients without either problem in a study by Dahlen on patients with obstructive lung disease [7]. Also, in a study by Ng on Singapore resident COPD patients FEV_1%predicted _alone was not able to predict the presence of anxiety and depression [9]. In a study by Mishima FEV_1 _did not correlate with the anxiety score and had only a borderline correlation with the depression score in COPD patients with long-term domiciliary oxygen therapy [22]. In concordance with our findings, dyspnoea correlated with both anxious and depressive symptoms.

In our data BODE index better explained the psychological consequences of COPD compared to the GOLD classification based on FEV_1%predicted _alone. Due to the incorporation of dyspnoea and exercise capacity the BODE index is a reliable predictor of objective COPD outcomes such as hospitalisation and survival [24,26]. On the one hand severe dyspnoea and reduced exercise capacity are obvious indicators for advanced lung disease. On the other hand our data show that they are also associated with symptoms of anxiety and depression, which themselves are independent predictors of objective COPD outcomes such as readmission and survival [6,7,9]. Therefore anxiety and depression might explain a part of the predictive power of the BODE index regarding objective COPD outcomes. It is unknown whether anxiety and depression remain independent predictors of clinical outcome of COPD, if the disease is staged by the BODE system. If so, these psychiatric co-morbidities might play a role in future classification systems of COPD. Anxiety and depression are aspects of COPD susceptible to both pharmacological and non-pharmacological treatment [10]. Specifically, psychotherapy reduces anxiety and depression in COPD [34]. Moreover, pulmonary rehabilitation improves depression, anxiety, dyspnoea and health status in patients with COPD [35,36].

Due to the cross-sectional design of the present study only associations can be assessed and causal inferences cannot be drawn. The dyspnoea score was the only factor associated with anxious symptoms in linear regression. It is quite evident that dyspnoea can cause anxiety. On the other hand, presence of anxiety might also aggravate the sensation of dyspnoea. Depressive symptoms were best explained by the dyspnoea score and the six minute walking distance. It is well imaginable that patients who suffer from breathlessness and whose exercise capacity is limited are at increased risk of depression. On the other hand, depressive symptoms might also worsen the sensation of dyspnoea and limit the effort during the walking test. Whether or not depression and anxiety are comorbidities in COPD, they influence the clinical outcome of COPD [6,7,9]. The small number of patients in GOLD stage I is a limitation of the study. However, these patients usually do not experience dyspnoea and are therefore unlikely to have consecutive anxiety or depression.

## Conclusion

In conclusion, anxious and depressive symptoms are common in patients with advanced COPD. The BODE index is superior to the GOLD classification for explaining anxious and depressive symptoms in COPD patients. Future classifications of COPD severity might include those psychological aspects, as they are potentially treatable aspects of the disease.

## Abbreviations

ATS: American Thoracic Society; AUROC: area under the receiver operator characteristic curve; BMI: body mass index; BODE: body mass index, obstruction, dyspnoea, exercise; CI: confidence interval; COPD: chronic obstructive pulmonary disease; ERS: European Respiratory Society; FEV1: forced expiratory volume in one second; FEV_1%predicted_: forced expiratory volume in one second in percent of the predicted value; GOLD: global initiative for chronic obstructive lung disease; HAD scale: hospital anxiety and depression scale; Kτ: Kendall's rank correlation coefficient; MMRC: Modified Medical Research Council dyspnoea scale; PFT: pulmonary function test; SPSS: Statistical package for the social sciences; χ^2^: chi squared

## Competing interests

The authors declare that they have no competing interests.

## Authors' contributions

GF performed the statistical analysis and wrote the manuscript. KK participated in the design of the study, created the questionnaires and performed patient interviews. SH conceived of the study, participated in its design and coordination and helped to draft the manuscript. OCB helped to draft the manuscript. All authors read and approved the final manuscript.
